# Systematic analysis of genes involved in oral cancer metastasis to lymph nodes

**DOI:** 10.1186/s11658-018-0120-2

**Published:** 2018-11-15

**Authors:** Xing’an Zhang, Lanfang Zhang, Xiaoyao Tan, Ying Lin, Xinsheng Han, Huadong Wang, Huawei Ming, Qiujiang Li, Kang Liu, Gang Feng

**Affiliations:** 1 Department of Stomatology, Nanchong Central Hospital, The Second Clinical Medical College of North Sichuan Medical College, Nanchong, Sichuan 637000 People’s Republic of China; 2 Institute of Tissue Engineering and Stem Cells, Nanchong Central Hospital, The Second Clinical Medical College of North Sichuan Medical College, No. 95, People’s south Road, Shunqing District, Nanchong, Sichuan 637000 People’s Republic of China; 3 Department of Burn and Plastic Surgery, Nanchong Central Hospital, The Second Clinical Medical College of North Sichuan Medical College, Nanchong, Sichuan 637000 People’s Republic of China; 4 Department of Science and Education, Nanchong Central Hospital, The Second Clinical Medical College of North Sichuan Medical College, Nanchong, Sichuan 637000 People’s Republic of China

**Keywords:** Oral cancer, Metastasis, CCND1, JUN, SPP1

## Abstract

Oral cancer remains a deadly disease worldwide. Lymph node metastasis and invasion is one of the causes of death from oral cancer. Elucidating the mechanism of oral cancer lymph node metastasis and identifying critical regulatory genes are important for the treatment of this disease. This study aimed to identify differentially expressed genes (gene signature) and pathways that contribute to oral cancer metastasis to lymph nodes. The GSE70604-associated study compared gene profiles in lymph nodes with metastasis of oral cancer to those of normal lymph nodes. The GSE2280-associated study compared gene profiles in primary tumor of oral cancer with lymph node metastasis to those in tumors without lymph node metastasis. There are 28 common differentially expressed genes (DEGs) showing consistent changes in both datasets in overlapping analysis. GO biological process and KEGG pathway analysis of these 28 DEGs identified the gene signature CCND1, JUN and SPP1, which are categorized as key regulatory genes involved in the focal adhesion pathway. Silencing expression of CCND1, JUN and SPP1 in the human oral cancer cell line OECM-1 confirmed that those genes play essential roles in oral cancer cell invasion. Analysis of clinical samples of oral cancer found a strong correlation of these genes with short survival, especially JUN expression associated with metastasis. Our study identified a unique gene signature – CCND1, JUN and SPP1 – which may be involved in oral cancer lymph node metastasis.

**Electronic supplementary material:**

The online version of this article (10.1186/s11658-018-0120-2) contains supplementary material, which is available to authorized users.

## Introduction

Oral cancer is a major devastating head and neck cancer subtype and is any cancerous tissue growth located in the oral cavity or oropharynx [1]. There are several types of oral cancers, but around 90% of oral cancers are squamous cell carcinomas. Oral cancer is the sixth most common malignancy in humans. Its incidence and mortality have also increased over the past decades. In 2013, oral cancer resulted in 135,000 deaths, up from 84,000 deaths in 1990 [2]. Oral cancer is characterized by poor prognosis and a low survival rate despite sophisticated surgical and radiotherapeutic modalities. The five-year survival rate is only 63% in the United States.

Metastasis is one of the main causes of oral cancer patients’ death. The process of oral cancer metastasis is a series of sequential and interdependent events involving detachment of cells from tumor tissue, increased cell motility and local invasion, angiogenesis, intravasation of invading cells into the vasculature or lymphatic systems, extravasation and subsequent deposition and proliferation at a second site. Oral cancer tends to spread primarily to the regional lymph nodes of the neck first before it spreads to remote sites. Lymph node metastasis is called locoregional metastasis and lung (or other organs) metastasis is called distant metastasis [3]. Early diagnosis of lymph node metastasis is important for improving clinical outcomes of oral cancer patients [4].

The clinical diagnosis of lymph node metastasis of oral cancer is currently based on imaging techniques and sentinel lymph node biopsy (SLNB) [5]. However, current imaging tests have been proven to be unreliable, especially in the detection of early nodal diseases. Localization of SLNB in patients with certain cancers such as floor-of-mouth carcinoma is difficult. The difficulties of detecting micrometastasis in frozen sections also limit SLNB as an adequate guide to clinical decision-making [6]. Therefore, better understanding of the lymphatic metastasis of oral cancer and developing new diagnostic strategies to predict the clinical behavior of the disease are desired [4].

Identifying reliable gene signatures or molecular biomarkers for oral cancer lymph node metastasis is highly valuable to use as a potential diagnostic or prognostic tool for routine clinical practice of oral cancer. Microarray analysis has been extensively used to examine the global gene expression changes in non-metastatic versus metastatic human tumor samples. Comparing the gene expression profiles of primary tumors from non-metastatic and metastatic tumor generated a list of genes whose expression was significantly different in these two groups. The list of genes is called “metastasis signatures”. Several “metastasis signatures” have been generated in oral cancer aimed at predicting lymph node metastasis [7–17]. However, defined “metastasis signatures” from different studies are highly variable, which hinders their translation to clinical applications. To date, none of the identified signatures or molecular biomarkers has been successfully implemented as a diagnostic or prognostic tool applicable to routine clinical practice of oral cancer.

In this study, in order to identify differentially expressed genes (gene signature) and pathways that contribute to oral cancer metastasis to lymph nodes, we designed a new bioinformatic analysis strategy. The GSE70604-associated study is aimed at identifying lymph node metastasis-associated genes and pathways in oral cancer by comparing gene profiles in lymph nodes with metastasis to those of normal lymph nodes. The GSE2280-associated study tried to achieve this goal by comparing gene profiles in squamous cell carcinomas of the oral cavity with lymph node metastasis to those in tumors without lymph node metastasis. Then overlapping analysis was performed to identify common DEGs (gene signature) and pathways in these two studies. We focused on those 28 common DEGs that show consistent changes in both datasets and defined a “metastasis signature”: CCND1, JUN and SPP1 for oral cancer lymph node metastasis. Both Jun and SPP1 are known molecules involved in cancer cell invasiveness and tumor metastasis. CCND1 (cyclinD1) is a known critical factor in cell cycle progression, but the function of CCND1 in human cancer cell migration/invasion is not sufficiently understood despite a recent study showing that it directly regulates the focal adhesion pathway and promotes R3327 rat prostatic tumor cell migration/invasion and tumor metastasis [18] and affects cell migration and invasion in breast cancer [18–21]. Our study identified a novel “focal adhesion”-related gene signature (CCND1, JUN and SPP1) that might be applicable for diagnosis of oral cancer metastasis to lymph nodes.

## Materials and methods

### Cell lines

OECM-1 is a human oral cancer cell line derived from gingival epidermoid carcinoma of a patient [22]. The cells were cultured in RPMI1640 supplemented with 10% fetal bovine serum (FBS), 2 mmol/ l glutamine, 100 units/mL penicillin and 100 μg/mL streptomycin in a humidified atmosphere of 95% air and 5% CO_2_ at 37 °C.

### siRNA interference

SPP1, JUN and CCND1 siRNA smart pool (Cat. L-009152-00-0005) was purchased from Dharmacon/Thermo Fisher Scientific. Transfection of the siRNA oligonucleotide duplexes was performed in a 6-well plate (1 × 10 ^5^ cells per well) with Lipofectamine 2000 (Invitrogen, Inc.), using the methods recommended by the manufacturer. Knockdown of SPP1, JUN and CCND1 with siRNA was examined 72 h after siRNA transfection through western blotting.

### Western blot

Cells were lysed in a lysis buffer containing 50 mmol/L TRIS-HCl, pH 7.4, 150 mmol/L NaCl, 0.5% NP40, 50 mmol/L NaF, 1 mmol/L Na_3_VO_4_, 1 mmol/L phenyl-methylsulfonyl fluoride, 25 μg/mL leupeptin, and 25 μg/mL aprotinin and clarified by centrifugation (14,000 g for 30 min at 4 °C). The protein concentration of the cell lysates was determined using the Bradford Coomassie blue method (Pierce Chemical Corp.). Whole-cell lysates were separated by sodium dodecyl sulfate (SDS)-PAGE and transferred onto nitrocellulose membrane. The membranes were blocked with PBS containing 5% (*w*/*v*) skim milk at 4 °C for 2 h, washed with PBST (PBS with 0.05% Tween-20), and then incubated overnight with primary antibody. After washing with PBST, the membrane was incubated with a second antibody at room temperature for 2 h, washed with PBST and then developed with the ECL system. The results of Western blot were analyzed with Odyssey software version 3.0.

### Cell invasion assay

Cell invasion potential was measured with a Boyden transwell chamber consisting of upper inserts with 8-μm-pore-size filter membranes at the bottom of the inserts and lower wells in 24-well cell culture plates (Corning Life Sciences). 20 μl of 1:6 diluted Matrigel (2–3 mg/ml protein) was added to the center of each cell well insert. Coated inserts were placed in an incubator to allow the Matrigel to solidify for 20–30 min. Cells (3.5 × 10^5^ cells in 0.2 mL) suspended in serum-free medium with 0.1% bovine serum albumin were seeded into the inserts of the chambers. The inserts were then placed over the wells filled with 0.5 mL of 10% FBS culture medium and incubated in a 37 °C incubator for 24 h. Cells that had not penetrated the filter membrane in the inserts were wiped off with cotton swabs, and the cells on the underside of the filter membrane were fixed and stained with the HEMA-3 kit (Fisher Diagnostics). Invaded cells were counted in a total of 10 fields for each sample under a microscope with the 10X objective and the stained cell number per field was calculated.

### Differentially expressed genes (DEG) analysis

Differentially expressed genes of the GSE2280-associated study and GSE2280-associated study were analyzed. The expression abundance (FPKM) value of each gene was estimated by running cufflinks [23] and the differentially expressed genes were assessed by cuffdiff. Statistically differentially expressed genes between two groups were those genes with logFC (fold change) > 0.45 and adjusted *p* value < 0.05. The adjusted p value was obtained through applying Benjamini and Hochberg’s (BH) false discovery rate correction on the original p value, and the fold change threshold was selected based on our purpose of focusing on significantly differentially expressed genes.

### Hierarchical clustering

Hierarchical clustering was conducted [24] to classify analyzed samples based on gene expression profiles. Hierarchical clustering using differentially expressed genes (DEGs) demonstrated the global gene expression patterns in the samples. In addition, the DEGs were further extracted and classified in specific biological processes (Gene Ontology terms) and KEGG pathways. The expression pattern of those DEGs was characterized and heat maps of the DEGs were classified in targeted biological processes or KEGG pathways using the R package.

### GO and KEGG pathway analysis

We used the R packages GO.db, KEGG.db and KEGGREST to detect Gene Ontology categories and KEGG pathways with significant enrichment in DEGs for comparison across all measured genes. The significantly enriched biological processes were identified by *p* value less than the threshold value 0.05. For the KEGG pathway, the p value was also set to less than 0.05.

## Results

### Identification of potential genes related to oral cancer metastasis to lymph nodes through screening GEO database

In order to find the key genes regulating oral cancer metastasis to lymph nodes, we screened the GEO (gene expression omnibus) database for GSE70604 (https://www.ncbi.nlm.nih.gov/geo/query/acc.cgi?acc=GSE70604) [25] and GSE2280 (https://www.ncbi.nlm.nih.gov/geo/query/acc.cgi?acc=GSE2280) [12] as shown in Additional file 1: Table S1 and Additional file 2: Table S3.

In GSE70604, the comparisons of gene expression profiles were made between lymph nodes with metastasis of oral squamous cell carcinoma (OSCC) and normal lymph nodes (comparison 1).

In GSE2280, the comparisons of gene expression profiles were made between primary tumors of OSCC which has lymph node metastasis and nonmetastatic primary OSCC without lymph node metastasis (comparison 2).

**Fig. 1:**
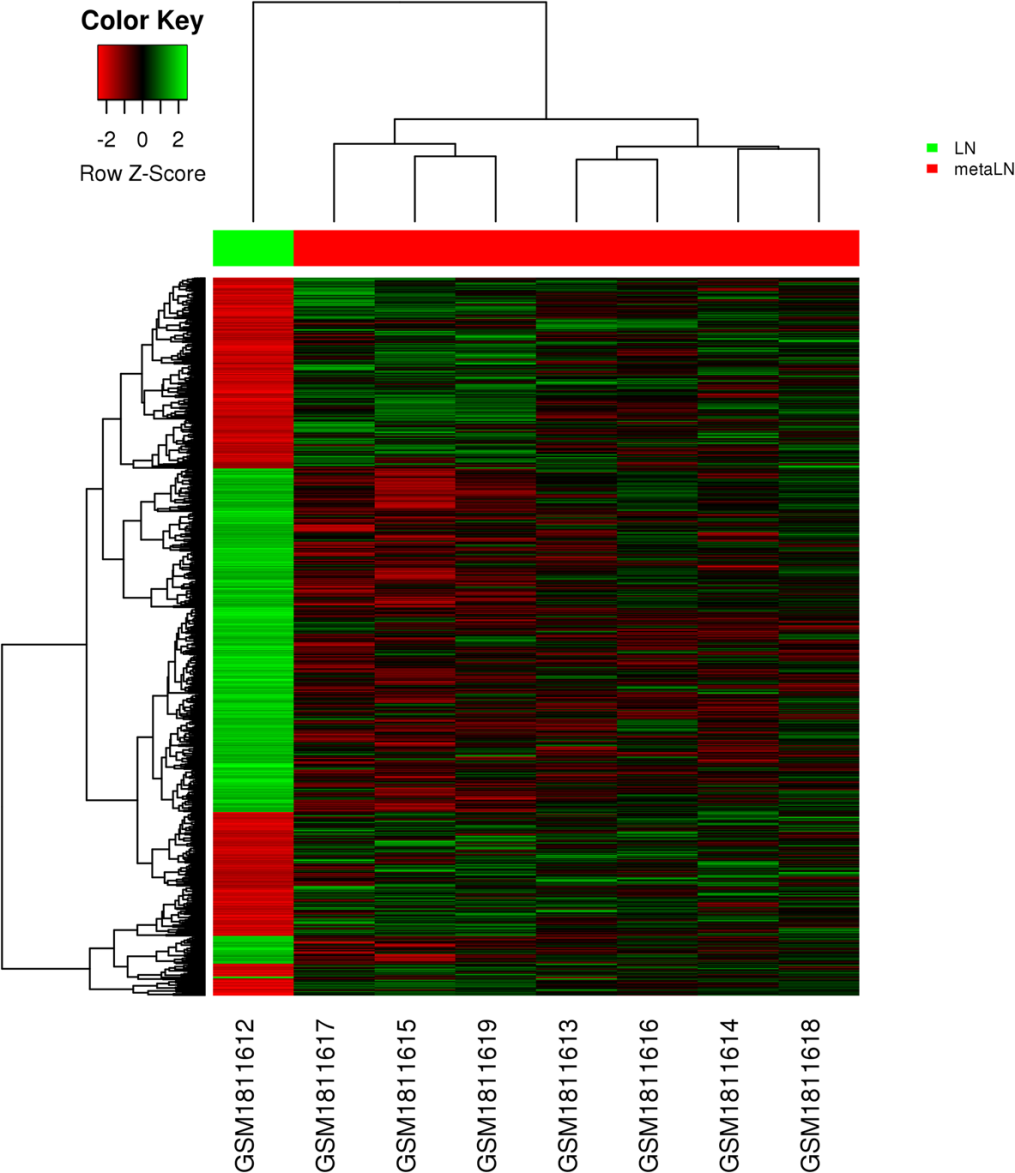
Heat map of distribution of DEGs in GSE70604. The comparisons of gene expression profiles were made between lymph nodes with metastasis of oral squamous cell carcinoma (OSCC) and normal lymph nodes

**Fig. 2:**
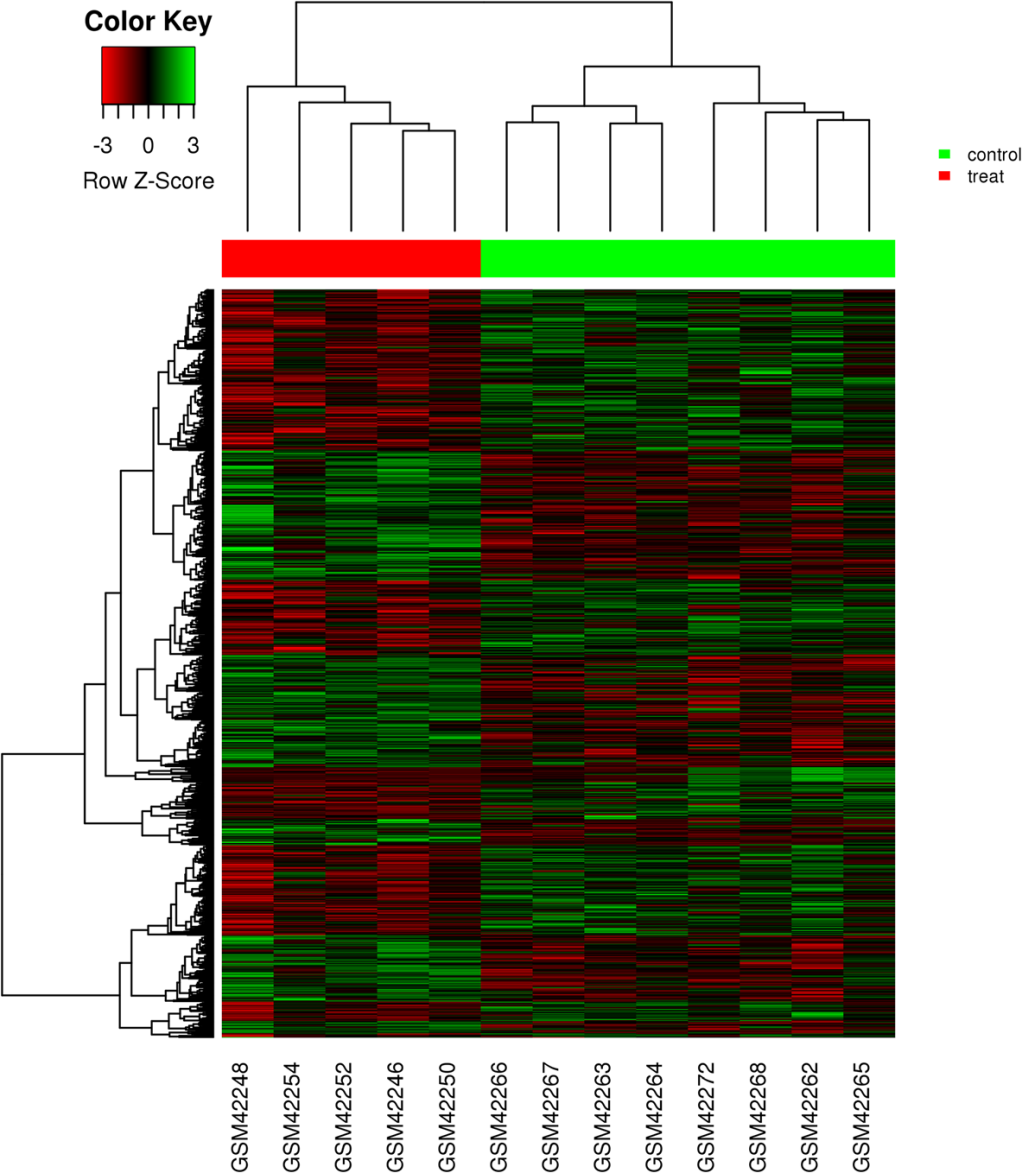
Heat map of distribution of DEGs in GSE2280. The comparisons of gene expression profiles were made between primary tumors of squamous cell carcinomas of the oral cavity (OSCC) which has lymph node metastasis and nonmetastatic primary OSCC without lymph node metastasis

Differentially expressed genes (DEGs) of both comparisons were obtained where lymph nodes with OSCC metastasis were compared to normal lymph nodes in comparison 1 and metastatic OSCC primary tumors were compared to non-metastatic OSCC primary tumors in comparison 2. Both comparisons had the |log(fold change)|(logFC) > 0.45 and *p* value < 0.05, indicating the overall changes as statistically significant. In comparison 1, gene expression of 7 lymph nodes with metastasis of OSCC was compared to that of a normal lymph node. Figure 1 shows the distribution of DEGs in comparison 1; we found that 1392 genes had expression changes (Additional file 1: Table S1). Among those genes, we identified 723 down-regulated genes (Additional file 3: Table S2) and 699 up-regulated genes (Additional file 2: Table S3). In comparison 2, gene expression of 5 primary tumors of OSCC with lymph node metastasis was compared to that of 8 non-metastatic primary tumors of OSCC. Figure 2 shows the distribution of DEGs in comparison 2; we found 890 genes that had expression changes (Additional file 4: Table S4). Among those genes, we identified 477 down-regulated genes (Additional file 5: Table S5) and 413 up-regulated genes (Additional file 6: Table S6). To identify potential genes related to oral cancer metastasis to lymph nodes, we then analyzed the overlapping DEGs in comparison 1 and comparison 2 and found 114 overlapping DEGs. Among those DEGs, 28 of 114 DEGs had a consistent change trend (consistent increase or decrease in both comparisons) (Additional file 7: Table S7) and the remaining 86 had an opposite change trend (Additional file 8: Table S8). The 28 genes with consistent changes in comparison 1 and comparison 2 are the potential genes related to oral cancer lymph node metastasis.

### GO-KEGG analysis of consistent overlapping DEGs

We further analyzed the 28 consistent overlapping DEGs by GO-KEGG analysis. GO biological process analysis indicated that these 28 genes were involved in 448 biological processes; Additional file 9: Table S9 shows the top 10 biological processes. KEGG pathway analysis showed that those genes mainly participated in 12 signaling pathways (Additional file 10: Table S10) and the top 3 KEGG signaling pathways are colorectal cancer, Toll-like receptor signaling pathway and Chagas disease. We also identified 7 interacting gene pairs among these 28 overlapping genes (Additional file 11: Table S11). Among those genes, JUN, ATF3 and FOS were presented as the key connecting nodes in the network of signaling pathways according to connectivity. JUN and FOS cooperatively participated in 9 common KEGG signaling pathway among the 12 KEGG pathways we identified. JUN, ATF3 and FOS were involved in 257, 115 and 183 GO-biological processes respectively.

### Definition of key regulatory genes involved in oral cancer metastasis to lymph nodes

Among 12 KEGG pathways we identified by analyzing the 28 consistent overlapping DEGs (Additional file 10: Table S10), we mainly focus on the focal adhesion pathway, which is important for cancer cell migration/invasion and tumor metastasis.

Three DEGs associated with the focal adhesion pathway in the 28 consistent overlapping DEGs are CCND1, JUN and SPP1. Both JUN and SPP1 are genes known to promote cancer cell invasiveness and tumor metastasis. CCND1 (cyclinD1), a critical gene regulating cell cycle progress, recently has been reported to directly regulate the focal adhesion pathway and promote R3327 rat prostatic tumor cell migration/invasion and tumor metastasis [18]. The direct involvement of CCND1 in human cancer cell migration/invasion is virtually unknown. Our study identified a novel focal adhesion-related gene signature (CCND1, JUN and SPP1) that might be important for oral cancer metastasis to lymph nodes.

### Gene signature CCND1, JUN and SPP1 are required for oral cancer cell invasion

**Fig. 3:**
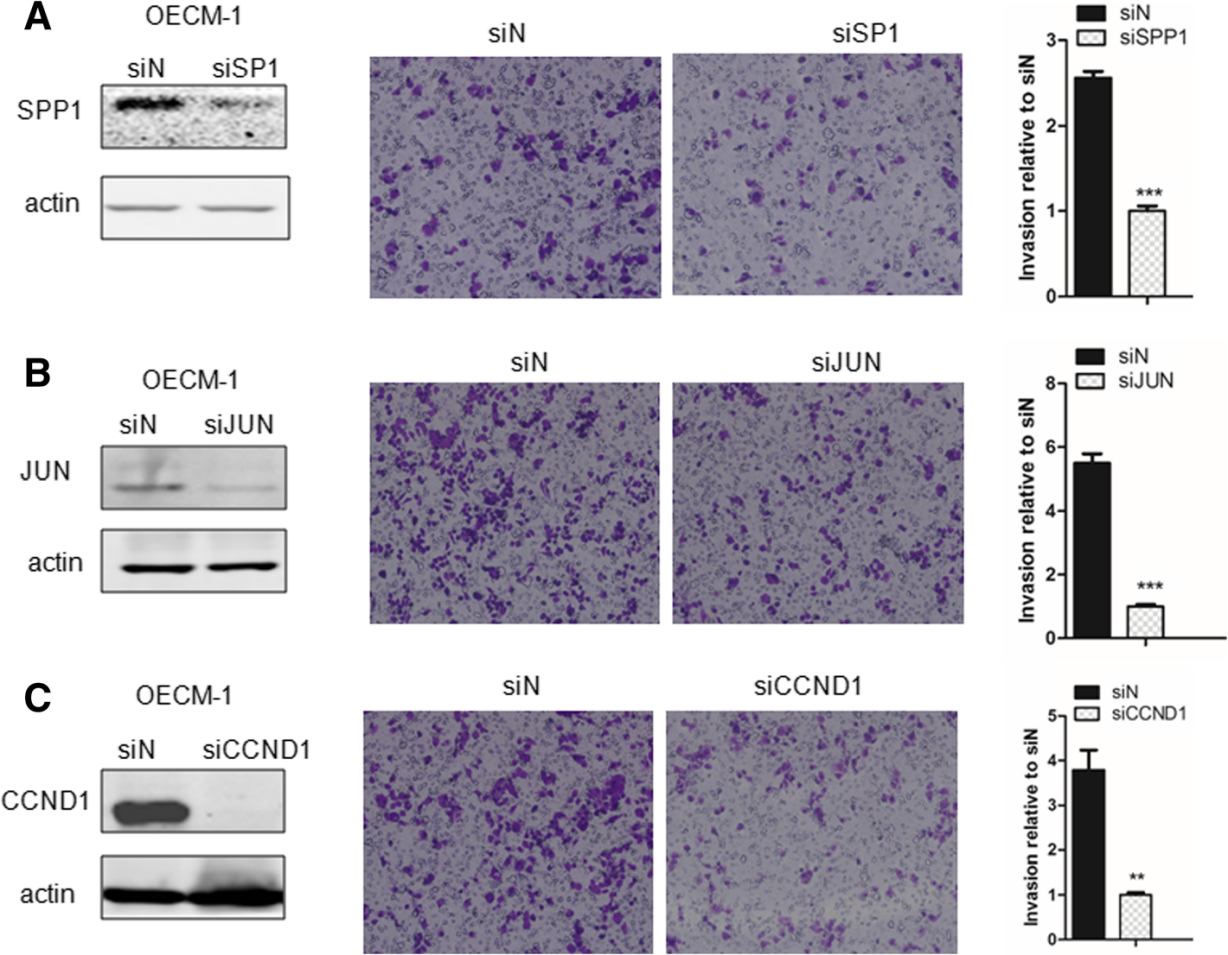
CCND1, JUN and SPP1 are involved in oral cancer cell invasion. **a** Human oral cancer cell line OECM-1 was transfected with SPP1 siRNA, SPP1 protein expression was examined through western blot, and alteration of cell invasion ability was examined and quantified as a histogram. **b** Human oral cancer cell line OECM-1 was transfected with JUN siRNA, JUN protein expression was examined through western blot, and alteration of cell invasion ability was examined and quantified as a histogram. **c** Human oral cancer cell line OECM-1 was transfected with CCND1 siRNA, CCND1 protein expression was examined through western blot, and alteration of cell invasion ability was examined and quantified as a histogram

To confirm the function of CCND1, JUN and SPP1 in oral cancer cell invasion, we knocked down expression of CCND1, JUN and SPP1 through siRNA in the human oral cancer cell line OECM-1 and examined the alteration of cell invasion ability. Deletion of CCND1, JUN and SPP1 expression in OECM-1 via siRNA interference significantly impaired cell invasion although in varying degrees (Fig. 3a-c). The result further supports the hypothesis that the focal adhesion-related gene signature (CCND1, JUN and SPP1) is important for oral cancer invasion.

### C- JUN expression is correlated with oral cancer metastasis in clinical practice

**Fig. 4:**
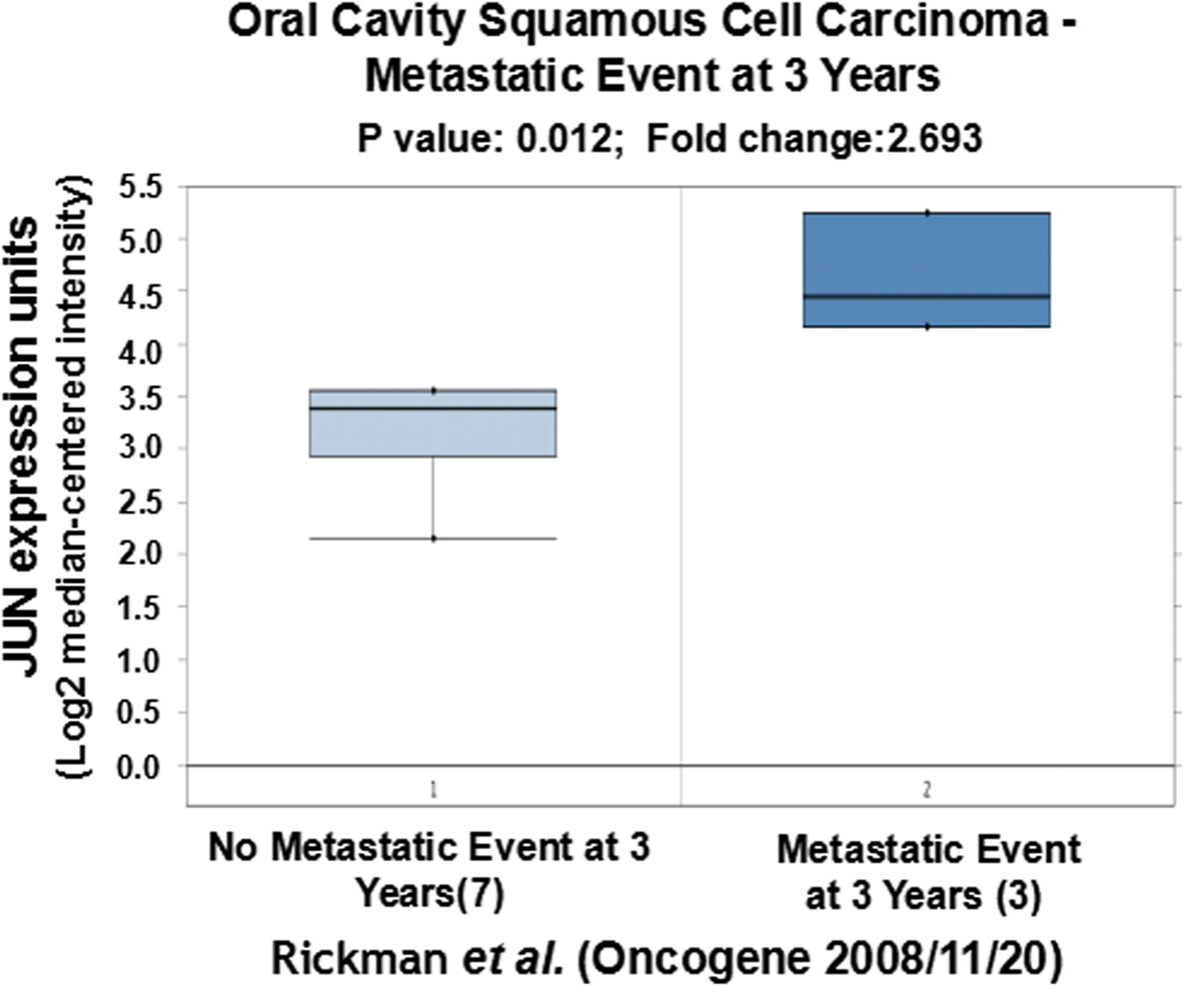
Comparison of JUN expression in oral cavity squamous cell carcinoma without metastatic events at 3 years and with metastatic events at 3 years

**Fig. 5:**
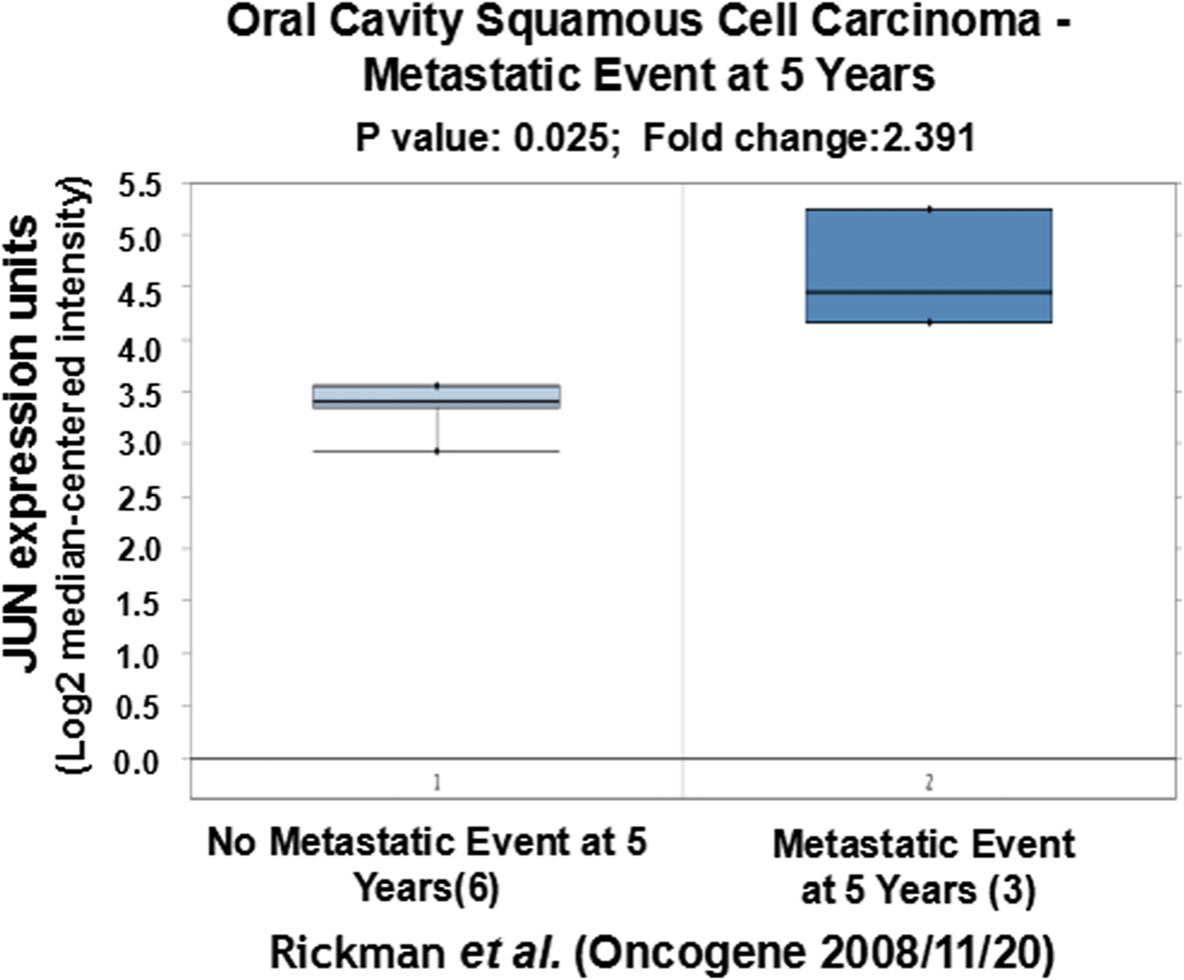
Comparison of JUN expression in oral cavity squamous cell carcinoma without metastatic events at 5 years and with metastatic events at 5 years

**Fig. 6:**
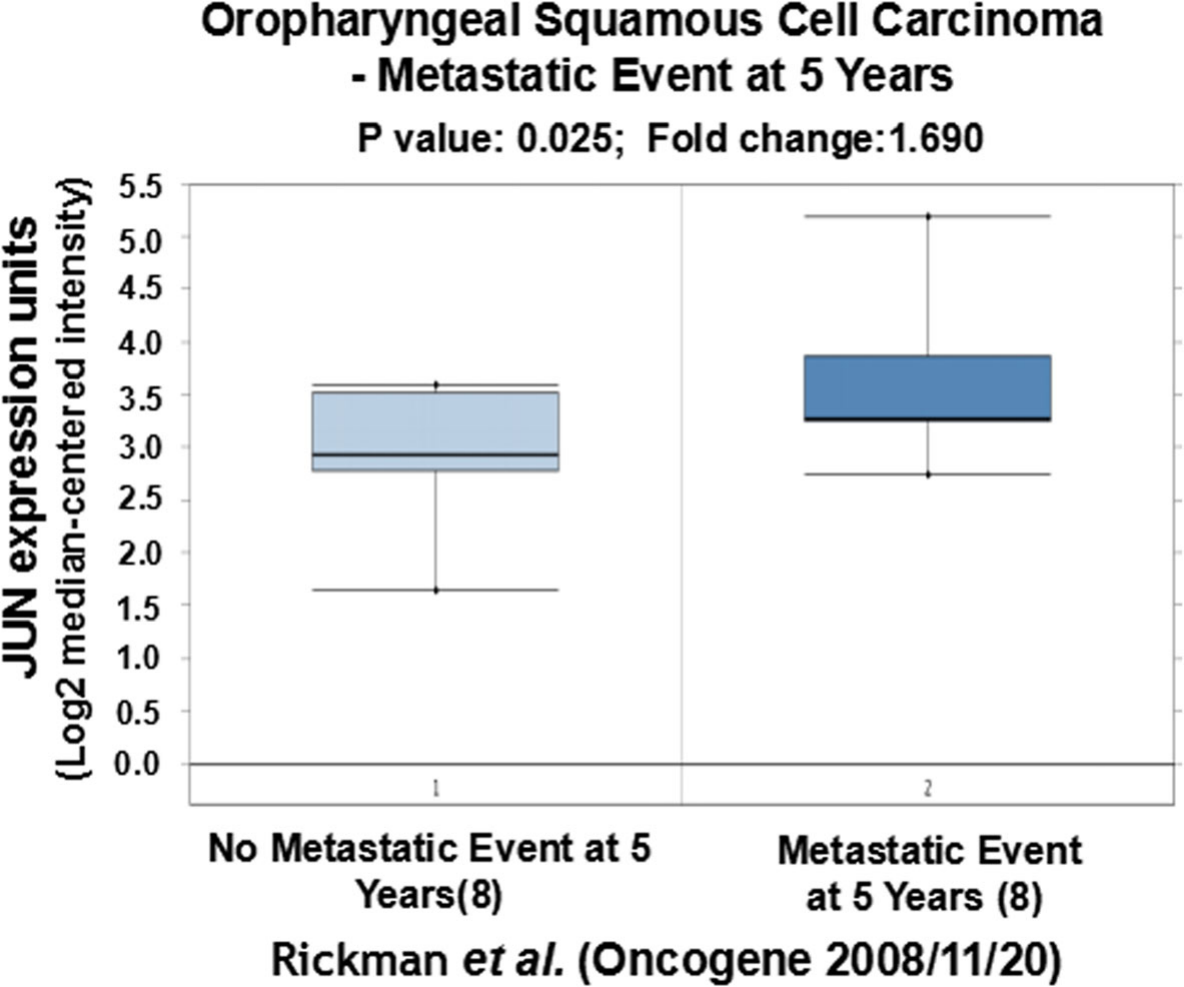
Comparison of JUN expression in oropharyngeal squamous cell carcinoma without metastatic events at 5 years and with metastatic events at 5 years

**Fig. 7:**
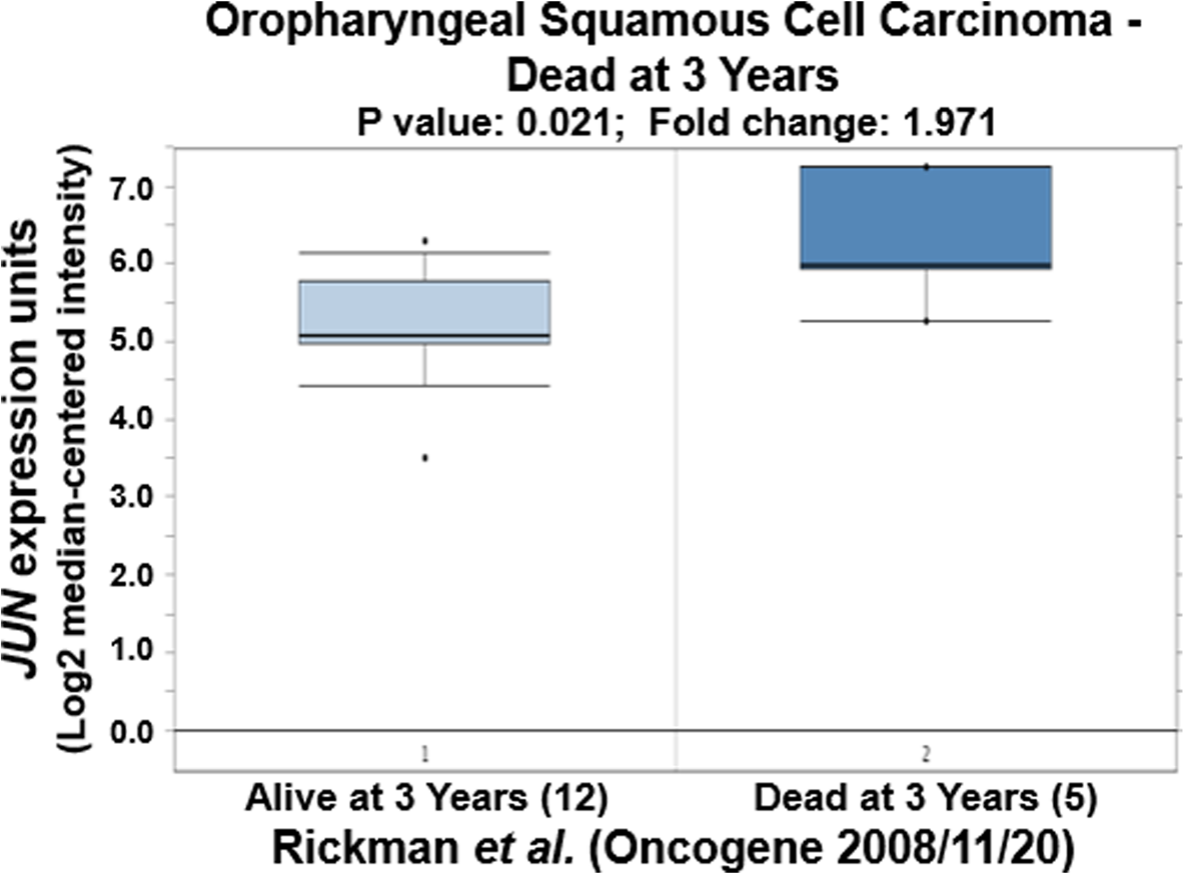
Comparison of JUN expression in oropharyngeal squamous cell carcinoma

**Fig. 8:**
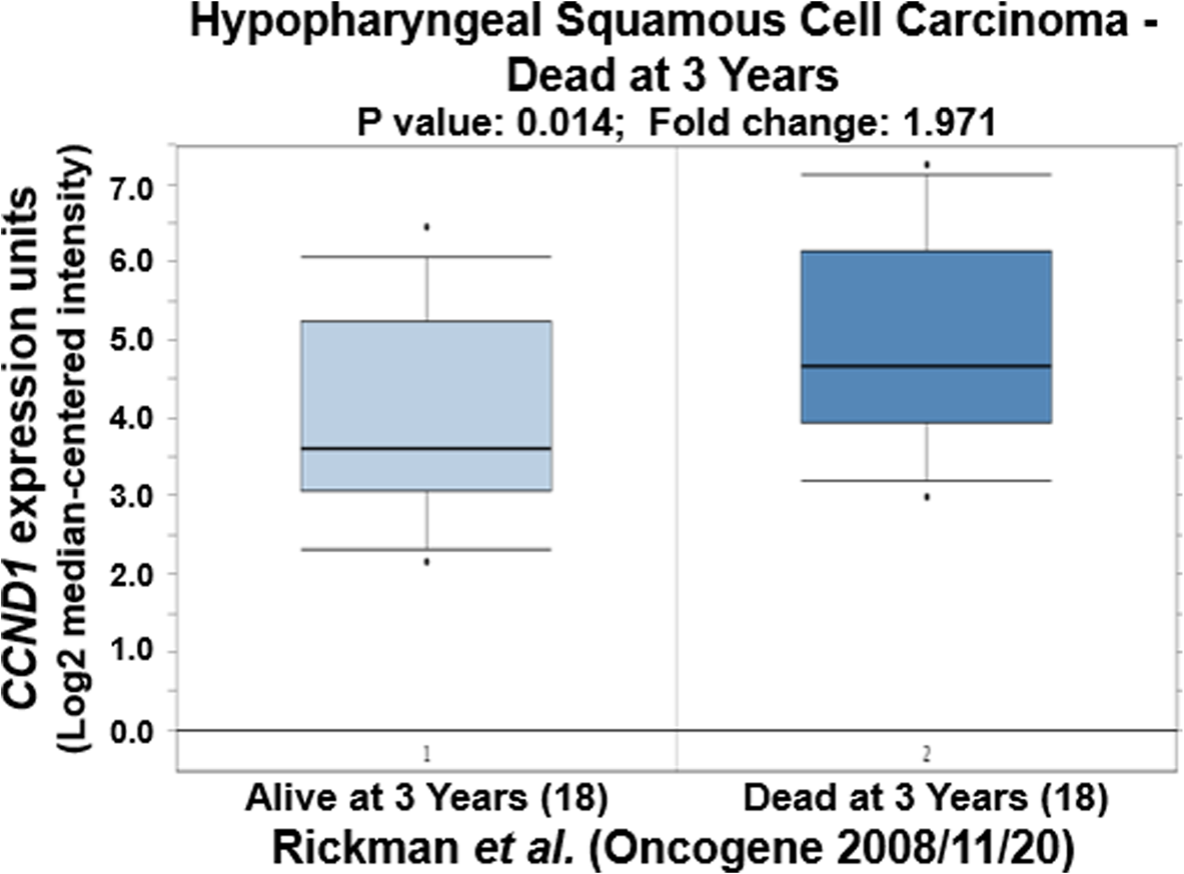
Comparison of CCND1 expression in hypopharyngeal squamous cell carcinoma

**Fig. 9:**
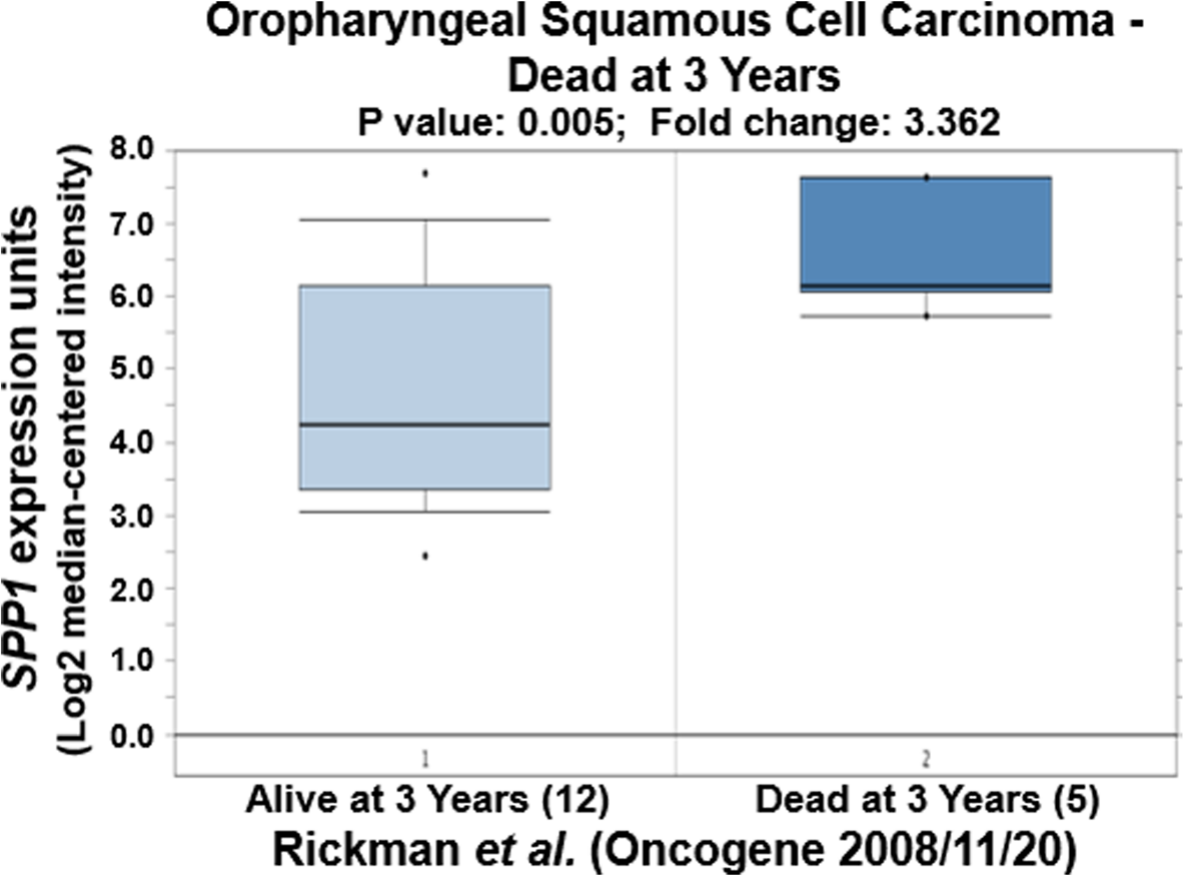
Comparison of SPP1 expression in oropharyngeal squamous cell carcinoma

We further tried to explore the correlation of expression of CCND1, JUN and SPP1 with oral cancer metastasis through statistically analyzing the clinic data in the literature. Based on the original data of Rickman’s work published in 2008 in Oncogene [26], we compared JUN expression in oropharyngeal squamous cell carcinoma in patients without metastatic events at 5 years with that of patients with metastatic events at 5 years [26]. Metastatic oropharyngeal squamous cell carcinoma had 1.69-fold higher JUN gene expression than non-metastatic oropharyngeal squamous cell carcinoma (*p* = 0.025). Figure 4 compares JUN expression in oral cavity squamous cell carcinoma in patients without metastatic events at 3 years with that of patients with metastatic events at 3 years [26]. Metastatic oral cavity squamous cell carcinoma had 2.69-fold higher JUN gene expression than non-metastatic oral cavity squamous cell carcinoma (*p* = 0.012) (Fig. 4). Figure 5 compared JUN expression in oral cavity squamous cell carcinoma in patients without metastatic events at 5 years than that of patients with metastatic events at 5 years [26]. Metastatic oral cavity squamous cell carcinoma had 2.39-fold higher JUN gene expression than non-metastatic oral cavity squamous cell carcinoma (*p* = 0.025) (Fig. 5). Figure 6 compares JUN expression in oropharyngeal squamous cell carcinoma in patients without metastatic events at 5 years with that of patients with metastatic events at 5 years [26]. Metastatic oropharyngeal squamous cell carcinoma had 1.69-fold higher JUN gene expression than non-metastatic oropharyngeal squamous cell carcinoma (p = 0.025) (Fig. 6). The results suggest that c-JUN is strongly correlated with oral cancer metastasis. We have not found similar gene expression data of CCND1 and SPP1 in oral cancer metastasis. However, we observed that higher expression of Jun or CCND1 or SPP1 was associated with short survival of oral squamous cell carcinoma patients (Figs. 7, 8 and 9).

## Discussion

Through bioinformatics analysis of the gene expression profile of oral cancer metastatic and non-metastatic lymph nodes and primary tumors, we identified a new oral cancer metastatic gene signature: CCND1, JUN and SPP1. The gene knockdown experiment on the oral cancer cell line and clinical data correlation analysis indicated strong association of this gene signature expression with oral cancer invasion and metastasis.

c-Jun is a subunit of transcription factor AP-1. Activated AP-1 increases the transcription of target genes and plays roles in cell division, proliferation, differentiation, apoptosis and so on. Although overexpression of c-Jun promotes invasion and metastasis of various tumors [27–29], it has not been reported in oral cancer. c-Jun plays a role during the initiation and progression of OSCC. High expression of c-Jun is associated with poor prognosis of OSCC [30]. Through a gene knockdown experiment in oral cancer cell line and clinical data analysis, our study confirmed that it is also required for oral cancer invasion and metastasis.

SPP1 (secreted phosphoprotein 1) encodes osteopontin (OPN). Osteopontin is a secreted non-collagenous, sialic acid rich, chemokine-like, matricellular phosphoglycoprotein that facilitates cell–matrix interactions and promotes tumor progression [31]. Osteopontin is a multifunctional cytokine regulating cell proliferation, survival, drug resistance, invasion, and stem-like behavior. Its aberrant expression and/or splicing is functionally responsible for many disease pathologies including cancer [31]. The expression of OPN was elevated in 95% of OSCC and can be used as a diagnostic marker for oral cancer [32]. It is also a poor prognostic factor in OSCC treated with cisplatin-based IC followed by CCRT [33]. However, its correlation with OSCC invasion and metastasis has not been studied anywhere else. The gene knockdown experiment in an oral cancer cell line and clinical data analysis showed that SPP1 is important for oral cancer invasion and metastasis in this study. Besides aberrant expression, the pattern of isoform expression (gene splicing) and post-translational modification are other SPP1 regulation methods. This regulation is cell-type specific and may influence the potential role of OPN in malignancy as a cancer biomarker [34] For example, invasive breast tumor cells generate three splice variants of OPN, while non-invasive breast cells express only the unspliced form or no OPN at all [35]. We do not know the splice variants of SPP1 in non-metastatic oral cancer and metastatic oral cancer with lymph node metastasis beside the expression difference, which deserves further investigation.

A recent study showed that CCND1 (cyclin D1) together with its binding partner CDK4 does not only simply act as a transcriptional regulator to control cell proliferation, but also controls cell adhesion, migration and metastasis under normal and pathological conditions. The focal adhesion component paxillin is a cytoplasmic substrate of CCND1·CDK4. This complex phosphorylates a fraction of paxillin specifically associated with the cell membrane, and promotes Rac1 activation, thereby triggering membrane ruffling and cell invasion in both normal fibroblasts and tumor cells [18]. Cytoplasmic CCND1 controls the migration and invasiveness of mantle lymphoma cells [36]. However, more evidence is needed to support the function of CCND1 in cell invasion and metastasis, especially in clinic samples. CCND1 is one of the DEGS with elevated expression in both metastatic lymph nodes and metastatic primary tumor of oral cancer in our analysis. The results suggested the important function of CCND1 in promoting oral cancer lymph node metastasis although the detained mechanism still needs to be explored.

In sum, this study identifies a unique gene signature – CCND1, JUN and SPP1 – which could be a new early biomarker for diagnosing oral cancer lymph node invasion and metastasis.

## Additional files

Additional file 1: **Table S1.** DEGs of GSE70604 Associated Study. (XLSX 106 kb)

Additional file 2: **Table S3.** Upregulated DEGs of GSE70604 Associated Study. (XLSX 57 kb)

Additional file 3: **Table S2.** Downregulated DEGs of GSE70604 Associated Study. (XLSX 59 kb)

Additional file 4: **Table S4.** DEGs of GSE2280 Associated Study. (XLSX 70 kb)

Additional file 5: **Table S5.** Downregulated DEGs of GSE2280 Associated Study. (XLSX 42 kb)

Additional file 6: **Table S6.** Upregulated DEGs of GSE2280 Associated Study. (XLSX 39 kb)

Additional file 7: **Table S7.** Consistent Overlapping DEGs. (XLSX 11 kb)

Additional file 8: **Table S8.** Opposite Overlapping DEGs. (XLSX 13 kb)

Additional file 9: **Table S9.** Top 10 GO Biological Processes of Consistent Overlapping DEGs. (XLSX 12 kb)

Additional file 10: **Table S10.** KEGG Pathways of Consistent Overlapping DEGs. (XLSX 10 kb)

Additional file 11: **Table S11.** Interacting Gene Pairs of Consistent Overlapping DEGs. (XLSX 9 kb)

## Abbreviations

CCND1: Cyclin D1
DEGs: Differentially expressed genes
GEO: Gene expression omnibus
OPN: Osteopontin
OSCC: Oral squamous cell carcinoma
SLNB: Sentinel lymph node biopsy
SPP1: Secreted phosphoprotein 1
